# Recognition of cyanobacteria promoters via Siamese network-based contrastive learning under novel non-promoter generation

**DOI:** 10.1093/bib/bbae193

**Published:** 2024-05-02

**Authors:** Guang Yang, Jianing Li, Jinlu Hu, Jian-Yu Shi

**Affiliations:** School of Life Sciences, Northwestern Polytechnical University, Xi’an, Shaanxi, 710072, China; School of Computer Science, Northwestern Polytechnical University, Xi’an, Shaanxi, 710072, China; School of Life Sciences, Northwestern Polytechnical University, Xi’an, Shaanxi, 710072, China; School of Life Sciences, Northwestern Polytechnical University, Xi’an, Shaanxi, 710072, China

**Keywords:** cyanobacteria promoters, Siamese network, negative sampling, motif analysis

## Abstract

It is a vital step to recognize cyanobacteria promoters on a genome-wide scale. Computational methods are promising to assist in difficult biological identification. When building recognition models, these methods rely on non-promoter generation to cope with the lack of real non-promoters. Nevertheless, the factitious significant difference between promoters and non-promoters causes over-optimistic prediction. Moreover, designed for *E. coli* or *B. subtilis*, existing methods cannot uncover novel, distinct motifs among cyanobacterial promoters. To address these issues, this work first proposes a novel non-promoter generation strategy called phantom sampling, which can eliminate the factitious difference between promoters and generated non-promoters. Furthermore, it elaborates a novel promoter prediction model based on the Siamese network (SiamProm), which can amplify the hidden difference between promoters and non-promoters through a joint characterization of global associations, upstream and downstream contexts, and neighboring associations w.r.t. k-mer tokens. The comparison with state-of-the-art methods demonstrates the superiority of our phantom sampling and SiamProm. Both comprehensive ablation studies and feature space illustrations also validate the effectiveness of the Siamese network and its components. More importantly, SiamProm, upon our phantom sampling, finds a novel cyanobacterial promoter motif (‘GCGATCGC’), which is palindrome-patterned, content-conserved, but position-shifted.

## INTRODUCTION

As a kind of prokaryotic bacteria, cyanobacteria cannot only produce oxygen by plant-like photosynthesis but also work as a green microbial cell factory for valuable natural products, chemicals and biofuels [1]. During these biological processes of cyanobacteria, promoters near transcription start sites (TSS) play an important role (i.e. recognize transcription units) in functional gene transcription [2]. Due to the specific binding of promoters to RNA polymerases, the identification of promoters helps understand how a gene is expressed [3]. However, since promoter recognition by biological assays is usually expensive and time-consuming, it is a difficult task to recognize promoters on a genome-wide scale.

Owing to a wealth of promoter sequences determined by high-throughput sequencing technology in the past two decades [4], computational methods have become a new promising approach to predict potential cyanobacteria promoters [5].

Existing methods can be roughly classified into three categories as follows. Scoring function-based methods exploit diverse promoter features (e.g. transcription binding sites, conserved sequence motifs) to construct a weight matrix or score matrix to rank newly given sequences [6–8]. Due to the weak characterization of common sequence features, these methods cannot achieve satisfactory prediction.

Machine learning-based (ML-based) methods are capable of recognizing potential sequences in a data-driven manner. They first represent promoter sequences as vectors based on various physicochemical properties and string features. Then, they explore traditional classifiers [e.g. support vector machines (SVM)] to discriminate whether a sequence is a promoter [9–11]. These methods effectively solve weak characterization of sequence features. However, their performance depends on the quality of hand-crafted features, which hardly capture promoter contextual features.

Compared to ML-based methods, deep learning-based (DL-based) methods [12–17] generally use neural networks to characterize promoter sequence context, leading to superior prediction due to better contextual feature characterization.

Although current ML-based and DL-based promoter prediction methods have achieved inspiring results, there are still some issues to be addressed. First, negative generation strategies in existing methods result in over-optimistic predictions, necessitating more effective approaches. Due to the prior absence of the common motif (e.g. −10 region Pribnow Box) in these generated non-promoters, the difference between them and promoters is implicitly significant to over-optimistic prediction. In contrast, as we observed in the literature [18], experimentally determined non-promoters are very similar to promoters. Existing negative sampling strategies cannot handle them well. Second, most existing computational methods for predicting promoters focus on *E. coli* and *B. subtilis*, with few methods designed for cyanobacterial promoters. In addition, compared to both *E. coli* and *B. subtilis*, cyanobacteria possess a nitrogen metabolism without ${\sigma}^N$-types (like ${\mathrm{\sigma}}^{54}$), which contains unobserved sequence motifs of cyanobacterial promoters [19]. Existing prediction methods cannot dig out novel potential motifs in cyanobacteria.

To address the disadvantages of normal negative sampling methods, this work proposes a novel negative sampling strategy, called phantom sampling, to generate non-promoters that are more suitable for cyanobacteria prediction tasks, which makes the model have better performance and generalization ability. Furthermore, considering the particularity of cyanobacterial promoter structure, this work elaborates on a novel promoter prediction model based on the Siamese network (SiamProm), which amplifies the sequence difference between promoters and non-promoters to distinguish them more easily. Overall, the main contributions of this work are as follows.

(i) We proposed a negative sampling method that is more suitable for promoter prediction tasks. It generates more real non-promoters, of which both GC contents and the Pribnow box look more like promoters. Such non-promoters enable prediction models to capture novel sequence features or potential motifs of cyanobacterial promoters.
(ii) SiamProm provides a novel multi-view feature representation method that can maximize the distinction between cyanobacterial promoters and non-promoters. Specifically, its representation learning module captures the globally important associations between pairwise k-mer tokens, upstream and downstream contexts of sequences, and the associations between neighboring tokens.
(iii) SiamProm, upon our phantom sampling, helps find a novel motif (‘GCGATCGC’) among cyanobacterial promoters. This motif is palindrome-patterned, content-conserved, but position-shifted.

## MATERIALS AND METHODS

### Promoter collection

We collected 13 705 experimentally validated promoter sequences of cyanobacteria (i.e. *Nostoc* sp. PCC 7120) from [20]. According to TSS, each promoter considers the core fragment from 60 bp upstream to 20 bp downstream regions. Moreover, since the original dataset may contain redundancy or noise [21], we applied CD-HIT-EST [22] with a cut-off value of 0.8 to exclude highly similar promoter sequences. Finally, the dataset contains 12 566 non-redundant promoters, taken as positive samples when building prediction models.

### Non-promoter construction

Building a prediction model requires positive samples (i.e. promoters) and negative samples (i.e. non-promoters). However, there are a few non-promoters annotated. Thus, non-promoter generation strategies were adopted.

The popular non-promoter generation strategy is sampling sequence fragments from the CDS region (CDS sampling) [5, 13, 23, 24]. In addition, some methods employ random generation [14] or partial substitution [12]. However, the generated non-promoters do not consist of significant common motifs (e.g. the −10 Pribnow Box), resulting in a significant difference between promoters and generated non-promoters. In other words, it is easy to distinguish promoters from non-promoters with even a simple model. For example, an SVM can achieve good prediction (88.54% accuracy) based on k-mer and GC contents under the CDS sampling strategy, while a DL-based method, DeePromoter [12], achieves 90.93% predicting accuracy (Table 2). Thus, such a factitious difference between promoters and non-promoters causes over-optimistic predictions.

More importantly, as we observed in the literature [18], experimentally determined non-promoters are very similar to the promoters regarding the conserved motif and GC content (Figure 3F and K). When the trained models under these sampling strategies meet real non-promoters, their performance becomes dramatically poor and non-robust. For example, the SVM achieves only 20.59% accuracy, while DeePromoter achieves 26.47% accuracy (Table 3).

**Figure 1:**
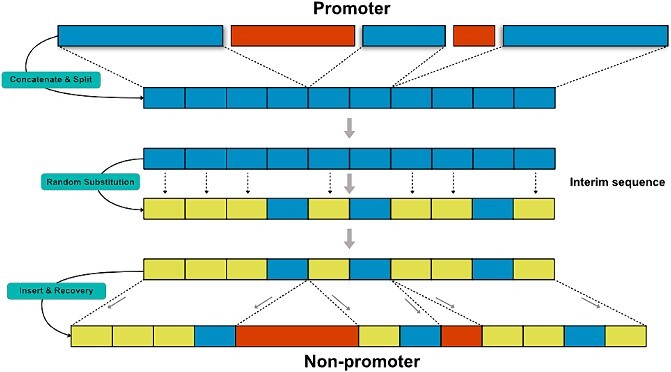
The process ofphantom sampling.

To cope with this issue, we designed a new non-promoter generation strategy called Phantom Sampling, which generates a corresponding non-promoter for each promoter. In the core idea of phantom sampling, non-promoters, compared with promoters, contain similar Pribnow Box, TSS and GC content but different bases in other regions. Figure 1 illustrates the steps involved in carrying out the phantom sampling procedure. First, based on each promoter (−60 to 20), both the Pribnow Box (−12 to −7) of the promoter and its TSS (−1 to 0) are fixed. Then, the remaining fragments are concatenated into an interim sequence and divided into 10 parts of equal length. After that, 7 out of the parts are randomized under the constraint that their average GC content has a <5% difference from that of the corresponding promoter. Finally, a non-promoter is constructed by returning these parts to their original positions in the promoter and merging them with the Pribnow Box and the TSS. The one-against-one phantom sampling boosts the prediction model to find hidden novel motifs different from well-known motifs (e.g. the Pribnow Box). Furthermore, it helps maintain an equal distribution of positive and negative samples during training.

### Datasets

Random generation, CDS sampling, partial substitution and phantom sampling are applied to generate four groups of non-promoters, respectively (Table 1). The number of non-promoters in each negative group is the same as that of promoters, where each sequence has a length of 81 base pairs. As suggested in [25], we constructed four datasets (DB1, DB2, DB3 and DB4) by combining promoters with each group of non-promoters accordingly. They are used to evaluate the prediction model under 10-fold cross-validation. In addition, we calculated the average GC content of non-promoters in each dataset [26]. We generated multiple groups of non-promoters based on phantom sampling to evaluate the robustness of SiamProm; the details are in Supplementary Table S2.

**Table 1:** Benchmark datasets

Datasets	Non-promoter generation	Avg. GC (%)	Promoter	Non-promoter
DB1	Random generation	49.94	12566	12566
DB2	CDS sampling	42.53		
DB3	Partial substitution	46.32		
DB4	Phantom sampling	37.41		
IND	CDS sampling	38.09	0	34

Moreover, although these datasets contain generated non-promoters, we collected 34 extra real non-promoters by literature searching as an independent testing set (IND). See also Supplementary Information. As observed (Figure 3F and K), they contain similar Pribnow Box, TSS and GC content as those of promoters, although they have no transcription function. Such a specific similarity between promoters and non-promoters poses a challenge to promoter recognition.

### Model architecture

**Figure 2:**
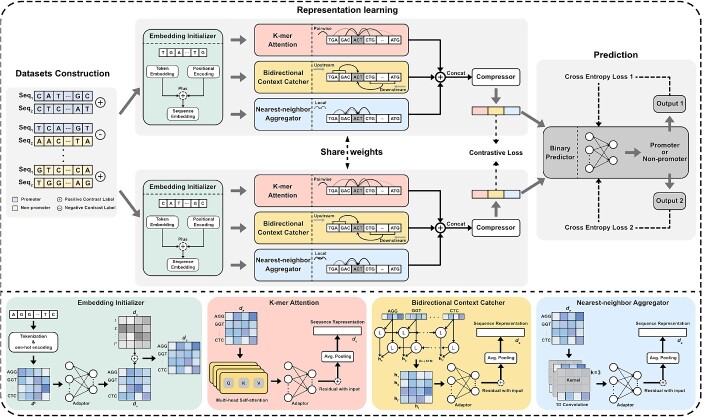
The architecture of SiamProm. The dataset construction module takes a pair of sequences as input, and the input labels include their labels and the contrastive labels. The embedding initializer splits sequences into a set of k-mer tokens and processes them into token embeddings with positional encodings. The k-mer attention module captures the globally important association between pairwise k-mer tokens. The bi-directional context catcher encodes the upstream and downstream contexts of sequences. The nearest-neighbor aggregator encodes the associations between neighboring tokens to capture the local features of sequences. The predictor acquires the representation of a sequence to distinguish whether it is a promoter or a non-promoter.

SiamProm is an end-to-end framework containing an elaborate Siamese network and a binary predictor. The former contains two identical sub-networks, which embed individual members of sequence pairs in parallel. If both sequences in the pair are promoters or non-promoters, we label the pair as a contrastively positive sample. If the sequence pair comprises a promoter and a non-promoter, it is considered a contrastively negative sample. The Siamese network learns sequence representations by distinguishing contrastively positive samples from contrastively negative pairs as well as possible. Each subnetwork comprises four modules: an embedding initializer, a k-mer attention module, a bi-directional context catcher, and a nearest-neighbor aggregator (Figure 2). The embedding initializer splits promoter sequences into a set of k-mer tokens and initializes them into one-hot encodings. Next, it passes the encodings through a fully connected layer and integrates them with token positional encodings. The attention module, containing a multi-head attention layer with residual connections, captures the globally important association between pairwise k-mer tokens. The bi-directional context catcher, implemented by a bi-LSTM, encodes upstream and downstream contexts of tokens, simultaneously characterizing the forward strand and the implicit reverse strand of sequences along with DNA. The nearest-neighbor aggregator, implemented by a 1D-convolution layer with residual connections, encodes the associations between neighboring tokens. In addition, each module, except for the embedding initializer, is followed by an adopter (i.e. MLP) to align token embeddings, which are further pooled (i.e. average-pooling) to obtain sequence embeddings. To obtain the final sequence embeddings, we concatenate all the sequence embeddings derived from the attention module, the context catcher, and the nearest-neighbor aggregator. For fast training, the compressor (i.e. MLP) is located after the embedding concatenation for dimensionality reduction. Finally, the promoter predictor (i.e. MLP) is trained to distinguish promoters from non-promoters. To ensure end-to-end training, the parameters of the representation learning module are frozen after updating, and the predictor module only updates its parameters. The mathematical theory and parameter settings of SiamProm are in the supplementary file.

### Prediction measure

To measure the prediction performance, this work adopts four commonly used classification metrics, including accuracy (Acc), sensitivity (Sn), specificity (Sp) and Matthew’s correlation coefficient (MCC). They are defined as follows: 


(1) \begin{align*} \begin{array}{c}\mathrm{Acc}=\dfrac{\mathrm{TP}+\mathrm{TN}}{\mathrm{TP}+\mathrm{TN}+\mathrm{FP}+\mathrm{FN}} \\[12pt]\mathrm{Sn}=\dfrac{\mathrm{TP}}{\mathrm{TP}+\mathrm{FN}} \\[12pt]\mathrm{Sp}=\dfrac{\mathrm{TN}}{\mathrm{TN}+\mathrm{FP}} \\[12pt]\mathrm{MCC}=\dfrac{\mathrm{TP}\times \mathrm{TN}-\mathrm{FP}\times \mathrm{FN}}{\sqrt{\left(\mathrm{TP}+\mathrm{FP}\right)\times \left(\mathrm{TP}+\mathrm{FN}\right)\times \left(\mathrm{TN}+\mathrm{FP}\right)\times \left(\mathrm{TN}+\mathrm{FN}\right)}}\end{array}\end{align*}

Specifically, TP is the count of accurately classified promoters, referred to as true positive; TN indicates the count of accurately classified non-promoters, referred to as true negative; FN denotes the count of promoters erroneously classified as non-promoters, called false negative and FP represents the count of non-promoters mistakenly classified as promoters, termed false positive. Sn is known as recall or the true positive rate, while Sp is known as the true negative rate. MCC is a correlation coefficient quantifying the global difference between correct and predicted labels.

## RESULTS

### Non-promoter generation analysis

**Figure 3:**
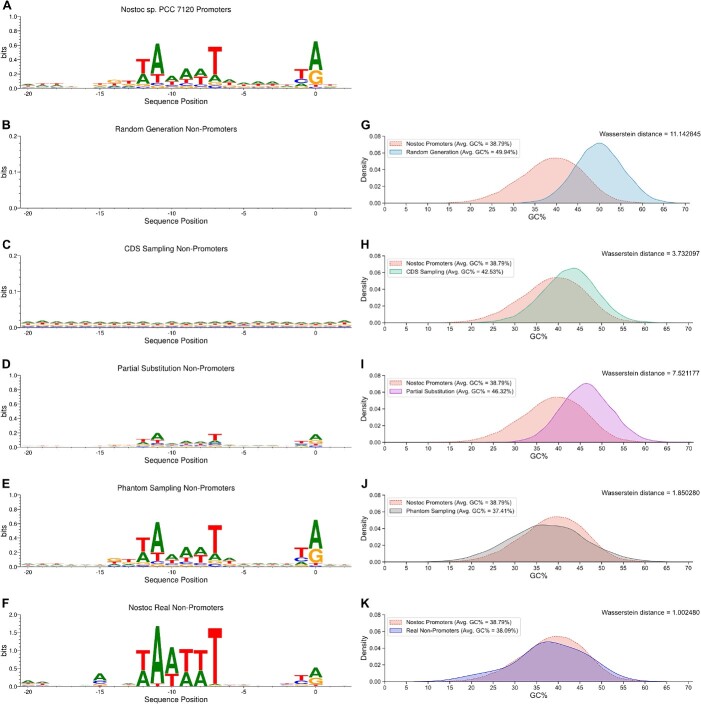
The results and analysis of non-promoter generation. (**A**–**F**) Seqlogo maps generated by promoters and different non-promoters. They display the distribution of bases at different positions in the sequences. (**G**–**K**) GC content distribution of promoters and non-promoters.

The difference between non-promoters generated by four strategies was investigated. First, the well-known motifs, including the −10 Pribnow Box and the TSS, were illustrated by SeqLogo [27] (Figure 3A–F). The illustration shows that an expected significant motif, ‘TAAAAT’, is inside the −10 Pribnow box, and a TSS motif is found in promoters. In contrast, neither random generation nor CDS sampling shows motifs (i.e. disordered), while partial substitution sampling weakly shows these motifs. Remarkably, non-promoters generated by our phantom sampling contain similar motifs as promoters. Moreover, those real non-promoters determined by biological assays exhibit similar motifs as promoters as well.

Then, the GC contents of generated non-promoters and real non-promoters were counted and compared with those of promoters. The statistical distribution difference between promoters and non-promoters was measured by Wasserstein distance (${d}_w$) [28]. The less the distance is, the more similar promoters and non-promoters are in terms of GC contents. The comparison shows that the non-promoters generated by random generation and partial substitution sampling significantly differ from promoters (i.e. ${d}_w^{\mathrm{rand}}=11.14$ and ${d}_w^{\mathrm{part}}=7.52,$ respectively). In addition, those generated by CDS sampling are moderately different from promoters (i.e. ${d}_w^{\mathrm{CDS}}=3.73$). In contrast, both the non-promoters generated by our phantom sampling and the real non-promoters show similar GC-content distributions as promoters (i.e. ${d}_w^{\mathrm{phan}}=1.85$ and ${d}_w^{\mathrm{real}}=1.00$, respectively).

The investigation demonstrates that traditional non-promoter generation strategies priorly imply a significant difference between promoters and non-promoters. This aforehand factitious difference would result in over-optimistic promoter predictions. However, real non-promoters show similar Pribnow box and TSS as observed. Thus, existing non-promoter generation strategies would not achieve a good prediction in real scenarios. Furthermore, since the non-promoters generated by our phantom sampling are similar to real non-promoters, it would be helpful to build a fairer and less unbiased predictor that focuses on finding unobserved motifs. See experimental validations for details in the next section.

### Method comparison over non-promoter generation strategies

To assess the performance of predicting methods w.r.t. non-promoter generations, we compared our SiamProm with four baseline methods, including one ML-based method, two DL-based methods [12, 14] and one ensemble learning method [13]. They are briefly introduced in the following.

(i) We designed an SVM-based method as the baseline. It was trained based on concatenating two popular handcraft features, including 3-mer (64-dimensional) and GC percentage (1-dimensional). We implemented the SVM by taking the radial basis function as the kernel function with a penalty factor set to 1.0 and setting the maximum iteration to 1000.
(ii) DeePromoter is an end-to-end DL-based model. It first uses a set of CNNs with size-varied convolutional kernels to characterize nucleotide base neighborhoods, then utilizes a Bi-LSTM to capture the long-distance association between nucleotide bases, and finally builds an MLP as the predictor. We retrain it on our datasets.
(iii) CyaPromBERT, a recently published DL-based method, utilizes BERT [29] to perform pre-training on genomes and tunes the pre-trained model on cyanobacteria promoters as the predictor. Due to the inner transformer encoders, it can capture contextual information in the promoter sequence. We tuned its pre-trained module to our datasets.
(iv) iPro-WAEL is an ensemble learning-based model composed of CNNs and random forests. Its CNNs utilize word embedding techniques to extract the sequence features, while its random forests utilize traditional sequence-based descriptors as features. We retrain it on our datasets.

The comparisons of our SiamProm with four baseline methods were run over four non-promoter generation strategies that are mentioned above.

In all the scenarios of generation strategies, the DL-based methods (i.e. SiamProm, iPro-WAEL, CyaPromBERT and DeePromoter) outperform the ML-based methods (i.e. SVM-based) since the latter relies on handcraft sequence features, which are inadequate to characterize the difference between promoters and non-promoters. Specifically, our SiamProm achieves the best prediction overall; the prediction performance of iPro-WAEL, CyaPromBERT and DeePromoter is in descending order; the basic SVM-based method gives the worst prediction metrics. The comparison demonstrates that SiamProm is more robust to various sampling methods because its Siamese network-based encoding can capture a more powerful representation of the difference between promoters and non-promoters.

On the other hand, the comparison between generation strategies shows that both random generation and CDS sampling give over-optimistic results, where even the SVM-based method can reach >90% accuracy with simple features (GC% and 3-mer) due to the aforehand factitious difference between promoters and generated non-promoters in these scenarios.

**Figure 4:**
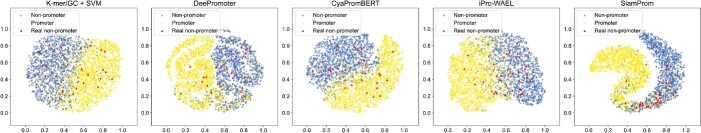
Latent spaces are built by five models under the phantom sampling. Only 2000 promoters and generated non-promoters are shown to give an easy-looking illustration. Real non-promoters are highlighted by red triangles.

Moreover, the prediction performances achieved by all the methods decrease in the scenarios of the partial substitution generation and the phantom generation, respectively, because the generated non-promoters contain the Pribnow box and the TSS, which can decrease the factitious difference. Note that our phantom generation can reduce the factitious difference better than the partial substitution generation. More results can be found in Table 3 and Figure 4 in the next section.

### Performance on real non-promoters

To further investigate which non-promoter generation strategy is appropriate in real scenarios, real non-promoters (Table 3) were input into the models built by random generation, CDS sampling, partial substitution and our phantom generation, respectively. As shown in Table 3, the popular generation strategies give rise to poor predictions over all the existing models. In detail, the base SVM-based model achieves poor predictions regarding random generation, CDS sampling and partial substitution. DeePromoter achieves poor prediction (<30% accuracy) in both random generation and CDS sampling but gives moderate prediction (i.e. 67% accuracy) in the case of partial substitution, where certain Pribnow boxes and TSS can be found in non-promoters. In addition, it is surprising that CyaPromBERT looks like a random guess in all the scenarios (i.e. 50% accuracy). The poor performance of CyaPromBERT may be because the fine-tuning data is different from the pre-training data, making it more difficult to identify real non-promoters. iPro-WAEL got 53% and 62% accuracy in random generation and CDS sampling while giving moderate prediction in partial substitution (i.e. 70% accuracy). In short, although inspiring performance is achieved in the case of cross-validation, three popular strategies of non-promoter generation result in dramatically reduced performance in the case of real non-promoters.

In contrast, our phantom sampling leads to significantly improved predictions over all the models. Compared to other generation strategies, the phantom generation helps the base SVM-based model achieve a moderate prediction (61.67%) with a 17.55%–44.02% improvement in accuracy. Similarly, it helps DeePromoter achieve 73.52% accuracy with a 5.88%–50.00% improvement. In addition, iPro-WAEL achieves 76.47% accuracy with a 5.89%–23.53% improvement. It also helps our SiamProm achieve the best prediction (85.29% accuracy) with 2.94%–26.47% improvement. The results demonstrate that our phantom generation is more appropriate to real scenarios.

On the other hand, our model can improve the prediction significantly over all the non-promoter generation strategies. In detail, it achieves 5.88%–41.17% improvement under random generation, 2.94%–44.11% improvement under CDS sampling, 5.89%–32.35% improvement under partial substitution and 11.76%–26.56% improvement under phantom generation. Especially under phantom generation, the performance of SiamProm on real non-promoters (88.23% in Table 3) is consistent with the performance on generated non-promoters (90.30% in Table 2). The comparison validates that our SiamProm significantly outperforms the existing methods.

**Table 2:** Performance comparison of five models with four negative generation methods on test datasets

Non-promoter generation	Model	Acc (%)	Sn (%)	Sp (%)	MCC
Random generation	K-mer/GC + SVM	90.22 (1.5e-16)	88.13 (4.0e-18)	92.30 (8.7e-16)	0.8051 (1.3e-08)
	DeePromoter	93.55 (3.7e-11)	93.10 (8.2e-09)	94.01 (1.0e-13)	0.8711 (2.1e-03)
	CyaPromBERT	95.13 (1.5e-07)	94.69 (1.2e-01)	95.58 (6.9e-10)	0.9027 (1.2e-02)
	iPro-WAEL	96.69 (2.4e-01)	**96.28** (1.0e+00)	97.10 (3.0e-06)	0.9339 (8.4e-01)
	Ours	**96.80**	95.08	**98.56**	**0.9367**
CDS sampling	K-mer/GC + SVM	88.54 (5.5e-16)	86.91 (8.4e-16)	90.18 (4.5e-16)	0.7713 (2.9e-06)
	DeePromoter	90.93 (1.1e-11)	88.79 (5.3e-14)	93.08 (3.2e-10)	0.8194 (2.2e-03)
	CyaPromBERT	92.04 (5.9e-06)	90.82 (3.3e-10)	93.26 (9.9e-08)	0.8411 (1.7e-04)
	iPro-WAEL	93.76 (4.8e-01)	**92.94** (1.0e+00)	94.59 (8.2e-03)	0.8754 (4.5e-01)
	Ours	**93.96**	92.53	**95.38**	**0.8797**
Partial substitution	K-mer/GC + SVM	79.22 (5.0e-19)	79.83 (2.7e-20)	78.60 (8.9e-20)	0.5844 (2.5e-07)
	DeePromoter	84.12 (1.6e-12)	83.94 (3.1e-14)	84.29 (1.1e-13)	0.6823 (2.9e-06)
	CyaPromBERT	86.11 (2.7e-05)	86.33 (5.0e-05)	85.88 (3.3e-07)	0.7221 (4.9e-03)
	iPro-WAEL	87.08 (1.8e-01)	86.47 (5.0e-05)	87.69 (3.2e-01)	0.7417 (1.2e-01)
	Ours	**87.57**	**87.39**	**87.74**	**0.7513**
Phantom sampling	K-mer/GC + SVM	78.33 (2.8e-22)	78.98 (3.0e-18)	77.69 (4.0e-22)	0.5667 (1.1e-09)
	DeePromoter	80.38 (1.6e-19)	80.27 (1.3e-15)	80.48 (8.0e-19)	0.6075 (1.4e-07)
	CyaPromBERT	82.48 (1.8e-17)	82.11 (1.1e-14)	82.86 (7.9e-17)	0.6497 (5.4e-07)
	iPro-WAEL	85.42 (6.8e-13)	84.97 (7.3e-08)	85.88 (4.5e-15)	0.7085 (2.2e-03)
	Ours	**88.74**	**87.20**	**90.30**	**0.7754**

*Note:* Boldface values represent the best values of the metrics under corresponding sampling.

**Table 3:** Performance on real non-promoters

Model	Random generation	CDS sampling	Partial substitution	Phantom sampling
K-mer/GC+SVM	17.65 (1.6e-30)	20.59 (1.3e-31)	44.12 (6.4e-28)	61.67 (1.1e-25)
DeePromoter	23.52 (1.6e-27)	26.47 (1.0e-30)	67.64 (2.9e-19)	73.52 (7.8e-22)
CyaPromBERT	47.05 (4.4e-20)	58.82 (1.3e-15)	52.94 (5.4e-26)	52.94 (5.1e-29)
iPro-WAEL	52.94 (1.6e-14)	61.76 (2.5e-11)	70.58 (3.2e-14)	76.47 (3.6e-21)
Ours	**58.82**	**64.70**	**76.47**	**88.23**

*Note:* Boldface values represent the best values of the metrics under corresponding sampling. Since all the real non-promoters are negative samples, the metric values in the table are Acc (%) or Sp (%).

**Table 4:** Ablation comparison

Module	Acc (%)	Sn (%)	Sp (%)	MCC	Acc^*^ (%)
w/o Siamese	82.66 (7.7e-17)	83.17 (2.3e-11)	82.14 (6.0e-19)	0.6531 (2.1e-06)	67.64 (5.3e-27)
w/o Attention	85.03 (8.1e-13)	85.22 (1.7e-06)	84.88 (9.3e-17)	0.7005 (1.9e-04)	79.41 (1.2e-18)
w/o BCC	87.23 (6.2e-09)	87.61 (9.7e-01)	86.91 (3.0e-13)	0.7445 (1.5e-03)	82.35 (1.5e-17)
w/o NNA	85.62 (3.3e-11)	87.39 (8.3e-01)	83.96 (6.0e-19)	0.7140 (2.1e-03)	76.47 (4.6e-20)
SiamProm	**88.74**	**87.20**	**90.30**	**0.7754**	**88.23**

*Note:* Boldface values represent the best values of the metrics under phantom sampling. Acc* represents the accuracy (Acc%) or specificity (Sp%) of the model on real non-promoters.

Furthermore, we illustrated how well promoters can be distinguished from non-promoters in the latent space. By *t*-distributed stochastic neighbor embedding (*t*-SNE) [30], under the phantom generation strategy, we mapped the sequence representation vector generated by the SVM, CyaPromBERT, DeePromoter and SiamProm into 2-dimensional latent spaces, respectively (Figure 4). These latent spaces show that our SiamProm has learned the difference between promoters (yellow) and non-promoters, including the generated non-promoters (blue) and the real non-promoters (red). The illustration demonstrates that our phantom generation can produce more appropriate non-promoters, which are more similar to real non-promoters. More importantly, the prediction model must learn other hidden sequence motifs because non-promoters show similar Pribnow boxes, TSS regions and GC contents as promotes (Figure 3F and K). See how SiamProm finds a novel motif in section Motif Analysis.

### Ablation study

In this section, we explored the contributions of crucial modules of SiamProm to the recognition of promoters through ablation studies.

Four SiamProm variants were constructed, and each masks a module in the representation learning stage. The first removed the whole Siamese network (denoted as w/o Siamese). The second one removed the attention module in the Siamese network (denoted as w/o attention). The third removed the bi-directional context catcher (denoted as w/o BCC). The last removed the nearest-neighbor aggregator (denoted as w/o NNA). The experiments were run in 10-fold cross-validation and an independent test on real non-promoters, respectively.

The comparison reveals that SiamProm outperforms other variants significantly (Table 4). The results demonstrate that the Siamese network is the most crucial module in our model. The underlying reason is that it can capture the difference between promoters and non-promoters. Its inner contrastive learning pulls within-class samples closer and pushes between-class samples farther away in the feature space. Moreover, three other modules in the Siamese network play untrivial roles in sequence embedding, respectively. Precisely, its k-mer attention module captures the globally important association between pairwise k-mer tokens. Its bi-directional context catcher encodes upstream and downstream contexts of sequences, while its nearest-neighbor aggregator encodes the associations between neighboring tokens to capture the local features of sequences.

In general, all these modules contribute to sequence representation, resulting in a good prediction.

### Motif analysis

**Figure 5:**
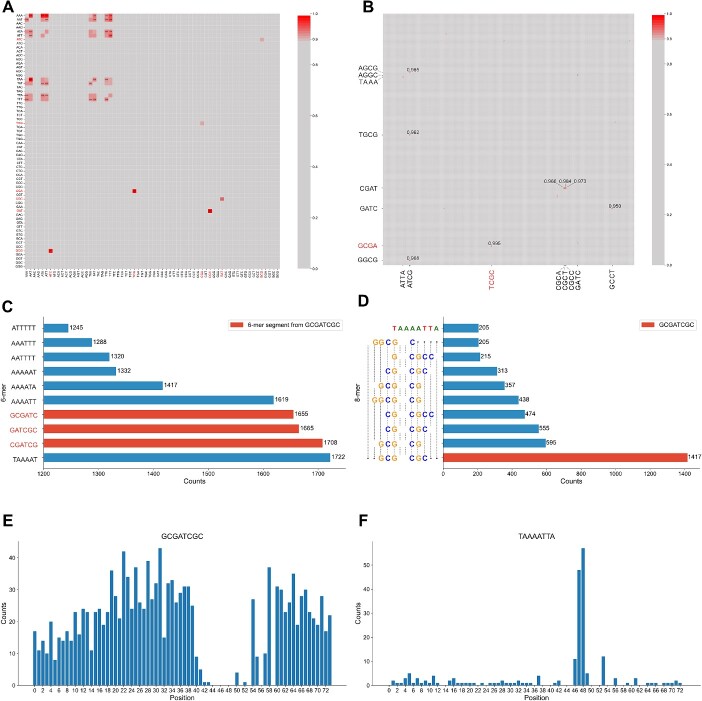
Motif analysis of promoters. (**A**) 3-mer attention map of promoters. We filtered 3-mer pairs with attention scores less than 0.9 and displayed specific attention scores with thresholds greater than 0.92. The highlighted tick labels show the 3-mer pairs of Rank-2–Rank-4. (**B**) 4-mer attention map of promoters. We filtered 4-mer pairs with attention scores less than 0.9 and displayed specific attention scores with thresholds greater than 0.95. The highlighted tick labels show the 4-mer pairs of Rank-1. (**C**) Top 10 6-mer fragments. (**D**) Top 10 8-mer fragments. *Y*-axis tick labels show a simple sequence alignment of Top 9 8-mer fragments. (**E**) Position distribution of 8-mer GCGATCTC. (**F**) Position distribution of cyanobacterial 8-mer TAAAATTA.

This section illustrates that our SiamProm can discover a novel potential motif among cyanobacterium promoter sequences. We leveraged the self-attention module of SiamProm (formula 4) to highlight the important k-mer token pairs. Technically, the attention scores of all promoter sequences w.r.t. k-mer tokens were averaged and normalized to obtain the global attention matrix (Figure 5A), where columns represent upstream tokens and rows represent downstream tokens. As we observed, the higher self-attention values between two tokens always indicate the nearer tokens along the sequence. Thus, a high attention score between two tokens possibly implies a longer consecutive token. For example, the high attention score between ‘CGA’ and ‘TCG’ reflects the token ‘CGATCG’.

By considering that a codon consists of three bases, conventional motif-finding methods always investigate 3-mer tokens in DNA sequences. After calculating the attention matrix of pairwise 3-mer tokens, we found that some token pairs have high attention scores ($\ge 0.9$), including ‘TAA-AAT’, ‘CGA-TCG’, ‘GAT-CGC’, ‘GCG-ATC’, ‘AAA-ATT’, ‘AAA-ATA’, ‘AAA-AAT’, ‘AAT-TTT’, ‘AAA-TTT’ and ‘ATT-TTT’. They indicate corresponding 6-mer tokens.

We further counted the occurrences of all possible 6-mer tokens, where the above 10 tokens are just ranked as the top 10 (Figure 5C). In detail, the result shows that the occurring number of ‘TAAAAT’ is the largest among all the tokens, and the tokens of Rank 5–Rank 10 are similar to ‘TAAAAT’, which consists of only adenine (A) and thymine (T). After investigating their occurring positions (Supplementary Figure S1 A–J), we found that such A/T-rich tokens correspond to the significant −10 motif of the cyanobacterium promoters [31]. Moreover, it is remarkable that three novel tokens, ‘CGATCG’, ‘GATCGC’ and ‘GCGATC’, are ranked as the second, third and fourth, respectively, in terms of occurring number. According to their positions, we found that they always occur in an overlapping way. This interesting finding implies a possible longer token (i.e. 4-mer token pairs).

In a similar manner, we found that several token pairs have high attention scores ($\ge 0.9$), including ‘GCGA-TCGC’, ‘AGCG-ATCG’, ‘CGAT-CGCT’ and ‘CGAT-CGCC’ (Fig. 5B). After counting the occurrences of their compositive 8-mer tokens (Fig. 5D), the top-10 rankings surprisingly show a possible palindrome 8-mer token, ‘GCGATCGC’. In detail, the occurring number of ‘GCGATCGC’ is ranked as the top-1 among all the tokens and nearly seven times that of the Rank-10 token ‘TAAAATTA’ (i.e. the extension of the well-known −10 motif). More importantly, the tokens of Rank-2–Rank-9 are highly similar to ‘GCGATCGC’, and their longest common sub-token, ‘CGATCG’, determined by multiple sequence alignment, is just ranked as the second in terms of 6-mer occurring number. Furthermore, by investigating their occurring positions (Supplementary Figure S2 A–H), we found that such a palindrome 8-mer token randomly occurs at any position along the promoter sequence but is excluded from the −10 position (Figure 5E and F). Therefore, we believe the palindrome token is a potential promoter motif with sequence conservation and position variance.

Moreover, we observed similar observations of palindrome tokens reported in the literature. For example, Xu *et al.* [32] explored that the restriction endonuclease MspI can recognize 5’-CCGG. Al-Attar *et al.* [33] found that clustered regularly interspaced short palindromic repeats (CRISPRs) are the hallmark of an ingenious antiviral defense mechanism in prokaryotes. Deng *et al.* [34] synthesized repetitive extragenic palindromic (REP) sequences in *E. coli* as an efficient mRNA stabilizer for protein production and metabolic engineering. Li *et al.* [35] discovered that the palindromic sequence Div2 (GTAAACATGTTTAC) could bind to specific transcription factors to regulate gene transcription, thereby affecting gene expression levels. Inspired by the above findings, we believe that the motif ‘GCGATCGC’ in cyanobacterium promoters has a certain function in promoter recognition. More biological assays are needed to validate its functions further.

## DISCUSSION

Despite the crucial role of cyanobacteria promoters, their identification is more difficult than that of coding regions [36]. The underlying reason is that cyanobacteria contain unknown motifs in addition to the well-known bacteria motif (i.e. Pribnow Box in −10 region), compared with other well-studied bacteria (e.g. *E. coli* or *B. subtilis*). In addition, some current works focus on detecting regulatory elements and structural units of chromatin [37–39], which helps researchers more comprehensively understand the function of species genes.

To assist biological assays, both ML-based methods and DL-based methods train a promoter recognizer (classifier) based on known promoters and non-promoters. In general, the non-promoters are generated by various generation strategies due to the lack of real non-promoters. Aiming to eliminate the factitious difference between promoters and generated non-promoters, this work proposes a novel non-promoter generation strategy, phantom sampling. The phantom sampling enforces the prediction model to find hidden novel motifs, different from well-known motifs, in other regions.

To dig out such hidden motifs, this work elaborates a novel promoter prediction model based on the Siamese network (SiamProm), which jointly characterizes sequences by capturing global associations, upstream and downstream contexts and neighboring associations w.r.t. k-mer tokens simultaneously. Moreover, the Siamese network amplifies the hidden difference between promoters and non-promoters by ensuring promoters or non-promoters are close to themselves while promoters and non-promoters are distant from each other. The comparison with other state-of-the-art methods in both classification and feature visualization demonstrates the superiority of SiamProm.

More importantly, our SiamProm, upon phantom sampling, finds a novel cyanobacterial promoter motif (‘GCGATCGC’), of which the occurring number is significantly larger than that of the Pribnow box. It also shows interesting properties, such as palindrome-patterned, content-conserved, but position-shifted. We believe that our SiamProm provides new insights into the structure of cyanobacteria promoters and can also be borrowed for promoters of other species. In the future, the function of the palindrome motif ‘GCGATCGC’ shall be investigated by biological assays. Moreover, this new structural information about cyanobacteria promoters can be helpful to novel promoter design in synthetic biology.

In addition, this study still has some limitations that are currently difficult to solve. In genomics, the biological functions of cyanobacteria are not only regulated by promoters, but it is also very important to detect the regulatory elements and structural units of chromatin. In future research, we will explore more of the intrinsic connections between other functional elements and promoters to explore the mechanism of cyanobacterial functions.

**Key Points**

- Phantom sampling generates more real non-promoters, of which both GC contents and the Pribnow box look more like promoters. Such non-promoters enable prediction models to capture novel sequence features or potential motifs of cyanobacterial promoters.
- SiamProm leverages contrastive learning to obtain discriminative sequence representations between promoters and non-promoters. Specifically, its k-mer attention module captures the globally important association between pairwise k-mer tokens. Its bi-directional context catcher encodes upstream and downstream contexts of sequences, while its nearest-neighbor aggregator encodes the associations between neighboring tokens to capture the local features of sequences.
- SiamProm, upon phantom sampling, helps find a novel motif (‘GCGATCGC’) among cyanobacterial promoters. This motif is palindrome-patterned, content-conserved, but position-shifted.

## Supplementary Material

Supplementary_Information_bbae193

## Data Availability

The source codes are available at https://github.com/Passion4ever/SiamProm.

## Author Biographies

**Guang Yang** is currently pursuing a PhD degree at the School of Life Sciences, Northwestern Polytechnical University, Xi’an, China. He is interested in deep learning and its application (e.g. promoter recognition, de novo protein design).

**Jianing Li** is currently working toward a PhD degree at the School of Computer Science, Northwestern Polytechnical University. Her research interests include molecular generation and drug discovery.

**Jinlu Hu** received her PhD degree from the Institute of Hydrobiology of the Chinese Academy of Sciences, where she is currently an associate professor at the School of Life Sciences, Northwestern Polytechnical University. Her research interest includes genomics and bioinformatics.

**Jian-Yu Shi** received his PhD degree from Northwestern Polytechnical University, Xi’an, China, where he is currently a professor. His research interests include bioinformatics, cheminformatics and artificial intelligence.
