# Supplementary material for: Recognition of cyanobacteria promoters via Siamese network-based contrastive learning under novel non-promoter generation
Source: Brief Bioinform. 2024 May 2;25(3):bbae193. doi: 10.1093/bib/bbae193 (PMC11066903; doi:10.1093/bib/bbae193)
Supplement: Supplementary_Information_bbae193 [file supplementary_information_bbae193.docx]

Supporting information for

**Recognition of Cyanobacteria Promoters via Siamese Network-based Contrastive Learning under Novel Non-promoter Generation**

Guang Yang^1^, Jianing Li^2^, Jinlu Hu^1,^* and Jian-Yu Shi^1,^*

^1^ School of Life Sciences, Northwestern Polytechnical University, Xi’an, Shaanxi, 710072, China

^2^ School of Computer Science, Northwestern Polytechnical University, Xi’an, Shaanxi, 710072, China

*Corresponding authors. Jinlu Hu, School of Life Sciences, Northwestern Polytechnical University, Xi’an 710072, China. Tel: +86 29 88460332; E-mail: hujinlu@nwpu.edu.cn; Jian-Yu Shi, School of Life Sciences, Northwestern Polytechnical University, Xi’an 710072, China. Tel: +86 29 88460332; E-mail: jianyushi@nwpu.edu.cn

**Sampling of pseudo-promoters**

We sampled 34 pseudo-promoters in the coding sequence of cyanobacterial genomes, with each sequence being 81 bp long. These sequences contain -10 consensus sequences in their relative positions compared to the promoter but have no transcriptional function, and they also share the same base preference in their positions near the promoter transcription site.

Specifically, we obtained the GeneBank file ([NC_003272](https://www.ncbi.nlm.nih.gov/nuccore/NC_003272.1)) of Nostoc sp. PCC 7120 used the coding region of each gene of the cyanobacteria as the sampling region and looked for a 13 bp fragment that extends from the cyanobacterial promoter -10 region to the transcription start site. The distance is determined. The pattern of this fragment is ‘WAWWWTNNNNNYR’ (Table S1), and the pattern is determined based on the base preference of the cyanobacterial promoter -10 motif. We ensured that these sequences sampled were not experimentally determined promoter sequences.

**Details of SiamProm**

**Sequence embedding initializer**

There exist diverse DNA encoding schemes, such as one-hot encoding [1], k-mer [2], the composition of k-spaced nucleic acid pairs (CKSNAP) [3], pseudo k-tuple nucleotide composition (PseKNC) [4], trinucleotide physicochemical properties (TPCP) [5], position weight matrix (PWM) [6] and Word2vec [7]. However, previous investigations demonstrate that they have no significant difference across different datasets and various models [8]. Thus, this work uses k-mer as the tokenization for promoter recognition.

The k-mer set indicates all possible nucleotide substrings with a length of $k$. Let $V$ be the k-mer token set, which contains $4^{k}$ unique tokens (i.e., $\left| V \right|=4^{k}$). It is prior to set $k=3$ because a codon includes three nucleotides [9,10]. In this case, $V=\left\{ AAA, AAC,...,TTT \right\}$. Based on $V$, an $l$-length nucleotide sequence $s='b_{1},\ldots,b_{l}'$ can be tokenized into an $l^{*}$-length token sequence $s^{*}='t_{1},\ldots,t_{l^{*}}'$, where $b_{i}\in\left\{ A,C,G,T \right\}$, $t_{i}\in V$ and $l^{*}=l-k+1$.

Then, the sequence is encoded as a matrix $\mathbf{S}\in\mathbb{R}^{l^{*}\times\left| V \right|}$, of which each row is the one-hot encoding vector w.r.t. token $t_{i}$. After that, the initial token representation $\mathbf{H}^{e}$ of $s$ is obtained by passing a fully-connected layer as $\mathbf{H}^{e}=\mathbf{S}\mathbf{W}_{s}$, where $\mathbf{W}_{s}\in\mathbb{R}^{\left| V \right|\times d_{e}}$ represents the learnable matrix and $d_{e}$ is the dimension of token embeddings.

Moreover, inspired by the Transformer [11], we employ the sine and cosine functions to encode the position of token $t_{i}$ into a unique representation $\mathbf{h}_{i}^{\mathrm{pos}}\in\mathbb{R}^{1\times d_{e}}$ as follows:

|  | $\mathbf{h}_{i}^{\mathrm{pos}}(2j)=sin\left( \frac{i}{r^{2j/d}} \right),\mathbf{h}_{i}^{\mathrm{pos}}(2j+1)=cos\left( \frac{i}{r^{2j/d}} \right),$ | (1) |
| --- | --- | --- |

where $j = 0,1,\ldots,N$, $N=d/2$ if $d$ is an even number or $N=\left( d-1 \right)/2$ if an odd number. The wavelengths form a geometric progression from $2\pi$ to $r\cdot2\pi$, where $r = 10000$ as suggested by [11]. Thus, the token positional encoding ($\mathbf{H}^{\mathrm{pos}}\in\mathbb{R}^{l^{*}\times d_{e}}$) of $s$ can be represented as a stack of $\left\{ \mathbf{h}_{i}^{\mathrm{pos}} \right\}$.

Finally, the initial token embedding of sequence $s$ is defined as:

|  | $\mathbf{H}=\mathbf{H}^{e}+\mathbf{H}^{\mathrm{pos}}$ | (2) |
| --- | --- | --- |

and it is input into three modules in parallel in the following sections.

**K-mer attention module**

The attention module captures globally important associations between pairwise k-mer tokens, which boosts finding potential motifs. It contains a multi-head attention layer with residual connections. The technical procedure is depicted in the following. Firstly, the initial token embedding ($\mathbf{H}$) is mapped into the Query **Q** $\boldsymbol{\in}\mathbb{R}^{l^{*}\times d_{k}}$ , the Key **K** $\boldsymbol{\in}\mathbb{R}^{l^{*}\times d_{k}}$ and the Value **V** $\boldsymbol{\in}\mathbb{R}^{l^{*}\times d_{v}}$ in parallel, where $\mathbf{Q}=\mathbf{H}\mathbf{W}^{Q}$, $\mathbf{K}=\mathbf{H}\mathbf{W}^{K}$, $\mathbf{V}=\mathbf{H}\mathbf{W}^{V}$ and $\mathbf{W}$ are learnable matrices accounting for specific linear projections. Then, the token representation is updated as $\mathbf{H}^{\mathrm{attn}}=\parallel_{r=1}^{h}\left( \mathbf{H}_{r}^{\mathrm{attn}} \right)$, where $\mathbf{H}_{r}^{\mathrm{attn}}$ represent its $r$-th attention head and defined as:

|  | $\mathbf{H}_{r}^{\mathrm{attn}}=\mathrm{softmax} \left( \frac{\mathbf{Q}\mathbf{K}^{T}}{\sqrt{d_{k}}} \right)\mathbf{V}.$ | (3) |
| --- | --- | --- |

Secondly, $\mathbf{H}^{\mathrm{attn}}\boldsymbol{\in}\mathbb{R}^{l^{*}\times{(r*d}_{v})}$ passes through an adaptor and then combines a residual connection [12] to enhance the token embeddings as follows:

|  | $\mathbf{H}^{\mathrm{attn}}\leftarrow\eta\left( \mathbf{H}^{\mathrm{attn}}\mathbf{W}^{a}+\mathbf{H} \right),$ | (4) |
| --- | --- | --- |

where $\eta\left( \cdot\right)$ represents the post-layer normalization function [13], $\mathbf{W}^{a}\boldsymbol{\in}\mathbb{R}^{{(r*d}_{v})\times d_{e}}$ is the learnable parameter of the adaptor, and $r$ represents the number of heads. Note that both the residual connection and the post-layer normalization are designed to handle the issues of gradient vanishing and exploding [12,13]. Lastly, the token embedding $\mathbf{H}^{\mathrm{attn}}$ passes through an average pooling layer to obtain the sequence embedding $\mathbf{v}^{\mathrm{attn}}$ as follows $\mathbf{v}^{\mathrm{attn}}=\frac{1}{l^{*}}\sum_{i=1}^{l^{*}} \mathbf{H}_{i}^{\mathrm{attn}}$,where $i$ represents the $i$-th token embedding.

**Bi-directional context catcher**

The bi-directional context catcher encodes upstream and downstream contexts of tokens, which characterizes a sequence (i.e., the forward strand) and its implicit reverse strand along with DNA simultaneously. It leverages Bi-LSTM to encode bi-directional token contexts.

With regard to time step t, let $\mathbf{i}_{t}\in\mathbb{R}^{1\times d_{l}}$ be the input gate, $\mathbf{f}_{t}\in\mathbb{R}^{1\times d_{l}}$ be the forget gate, $\mathbf{o}_{t}$ be the output gate, $\mathbf{g}_{t}\in\mathbb{R}^{1\times d_{l}}$ be the memory cells, $\mathbf{c}_{t}\in\mathbb{R}^{1\times d_{l}}$ be the memory cell state, $\mathbf{h}_{t}\in\mathbb{R}^{1\times d_{e}}$ be the input embedding (the $\mathbf{H}$’s t-th row corresponding to the t-th token), and $\mathbf{z}_{t}\in\mathbb{R}^{1\times d_{l}}$ be the hidden state, where $\mathbf{z}_{0}$ is a randomized vector. According to the LSTM, the updating rules of the hidden state for the forward strand can be defined as follows:

|  | $\mathbf{i}_{t}=\sigma\left( \mathbf{h}_{t}\mathbf{W}_{i}^{h}+\mathbf{z}_{t-1}\mathbf{W}_{i}^{z}+\mathbf{b}_{i} \right)$  $\mathbf{f}_{t}=\sigma\left( \mathbf{h}_{t}\mathbf{W}_{f}^{h}+\mathbf{z}_{t-1}\mathbf{W}_{f}^{z}+\mathbf{b}_{f} \right)$  $\mathbf{o}_{t}=\sigma\left( \mathbf{h}_{t}\mathbf{W}_{o}^{h}+\mathbf{z}_{t-1}\mathbf{W}_{o}^{z}+\mathbf{b}_{o} \right)$  $\mathbf{g}_{t}=\tanh\left( \mathbf{h}_{t}\mathbf{W}_{g}^{h}+\mathbf{z}_{t-1}\mathbf{W}_{g}^{z}+\mathbf{b}_{g} \right)$  $\mathbf{c}_{t}=\mathbf{f}_{t}⨀\mathbf{c}_{t-1}+\mathbf{i}_{t}⨀\mathbf{g}_{t}$  $\mathbf{z}_{t}=\mathbf{o}_{t}⨀\tanh\left( \mathbf{c}_{t} \right)$ | (5) |
| --- | --- | --- |

where $\mathbf{W}_{\boldsymbol{*}}^{h}\in\mathbb{R}^{d_{e}\times d_{l}}$, $\mathbf{W}_{\boldsymbol{*}}^{z}\in\mathbb{R}^{d_{l}\times d_{l}}$ and $\mathbf{b}_{\boldsymbol{*}}\in\mathbb{R}^{1\times d_{l}}$ are the weight matrices and the biases w.r.t. $\mathbf{i}_{t}$, $\mathbf{f}_{t}$, $\mathbf{o}_{t}$, and $\mathbf{g}_{t}$ respectively, and $\sigma$ denotes the sigmoid function and $⨀$ represents the Hadamard product. LSTM updates the *t*-th token embedding by $\mathbf{z}_{t}$. After $n$ steps (*n* is the number of tokens), all the hidden states are stacked as the token embedding matrix $\mathbf{Z}_{f}^{\mathrm{lstm}}=[\mathbf{z}_{1};\mathbf{z}_{2};\ldots;\mathbf{z}_{n}]\in\mathbb{R}^{l^{*}\times d_{l}}$.

Considering the implicit reverse strand of DNA, we also perform an extra reverse LSTM to obtain the token embedding matrix $\mathbf{Z}_{r}^{\mathrm{lstm}}\in\mathbb{R}^{l^{*}\times d_{l}}$. Thus, the final sequence embedding matrix $\mathbf{H}^{\mathrm{lstm}}$ is their concatenation $\mathbf{H}^{\mathrm{lstm}}=\left[ \mathbf{Z}_{f}^{\mathrm{lstm}},\mathbf{Z}_{r}^{\mathrm{lstm}} \right]$. Such a bi-directional embedding encodes upstream and downstream contexts of tokens.

Similar to $\mathbf{H}^{\mathrm{attn}}$ in the previous section, $\mathbf{H}^{\mathrm{lstm}}$ passes through an adaptor and then combines a residual connection [12] to enhance the token embeddings as follows:

|  | $\mathbf{H}^{\mathrm{lstm}}\leftarrow\eta\left( \mathbf{H}^{\mathrm{lstm}}\mathbf{W}^{a}+\mathbf{H} \right),$ | (6) |
| --- | --- | --- |

where $\eta\left( \cdot\right)$ represents the post-layer normalization function [13] and $\mathbf{W}^{a}\boldsymbol{\in}\mathbb{R}^{(2{*d}_{l})\times d_{e}}$ is the learnable parameter from the adaptor. An average pooling layer is further located to turn $\mathbf{H}^{\mathrm{lstm}}$ into the sequence embedding $\mathbf{v}^{\mathrm{lstm}}$ as follows $\mathbf{v}^{\mathrm{lstm}}=\frac{1}{l^{*}}\sum_{i=1}^{l^{*}} \mathbf{H}_{i}^{\mathrm{lstm}}$, where $i$ represents the $i$-th token embedding.

**Nearest-neighbor aggregator**

The nearest-neighbor aggregator, implemented by a 1D-convolution layer with residual connections, encodes the associations between neighboring tokens. In brief, a 1D-convolution on token embeddings can be described as:

|  | $\mathbf{H}^{\mathrm{conv}}=\mathbf{H}\star\mathbf{W}^{c}+\mathbf{b}^{c},$ | (7) |
| --- | --- | --- |

where $\star$ represents the convolution operator, $\mathbf{W}^{c}\boldsymbol{\in}\mathbb{R}^{d_{e}\times d_{c}}$ represents the weight matrix of the convolutional kernel, and $\mathbf{b}^{c}\boldsymbol{\in}\mathbb{R}^{d_{e}\times d_{c}}$ represents the bias term of the convolution. The convolution block aggregates local neighboring information of a token in the sequence.

Again, $\mathbf{H}^{\mathrm{conv}}$passes through an adaptor and then is combined with a residual connection [12] to enhance the token embeddings as follows:

|  | $\mathbf{H}^{\mathrm{conv}}\leftarrow\eta\left( \mathbf{H}^{\mathrm{conv}}\mathbf{W}^{a}+\mathbf{H} \right),$ | (8) |
| --- | --- | --- |

where $\eta\left( \cdot\right)$ represents the post-layer normalization function [13] and $\mathbf{W}^{a}\boldsymbol{\in}\mathbb{R}^{d_{c}\times d_{e}}$ is the learnable parameter from the adaptor. We use "same padding" to pad the sequence for the consistency of feature dimensions. In the same way, as those in previous sections, an average pooling layer turns $\mathbf{H}^{\mathrm{conv}}$ into the sequence embedding by $\mathbf{v}^{\mathrm{conv}}=\frac{1}{l^{*}}\sum_{i=1}^{l^{*}} \mathbf{H}_{i}^{\mathrm{conv}}$, where $i$ represents the $i$-th token embedding.

**Compressor module**

Naturally, all the embeddings of a sequence output by the k-mer attention module, the bi-directional context catcher, and the nearest-neighbor aggregator are concatenated into a higher dimensional embedding $\mathbf{v}^{\mathrm{concat}}=\parallel\left( {{\mathbf{v}^{\mathrm{attn}}\mathbf{,v}}^{\mathrm{lstm}}\mathbf{,v}}^{\mathrm{conv}} \right)$. To speed up the training, the compressor module, implemented by an MLP, performs a dimensionality reduction of sequence embeddings as follows:

|  | $\mathbf{v}=\mathbf{v}^{\mathrm{concat}}\mathbf{W}^{a}\boldsymbol{+}\mathbf{b}^{a}$ | (9) |
| --- | --- | --- |

where $\mathbf{W}^{a}\boldsymbol{\in}\mathbb{R}^{(3*d_{e})\times d}$ is the weight matrix of the compressor, and $\mathbf{b}^{a}\boldsymbol{\in}\mathbb{R}^{1\times d}$ is the bias.

So far, members in sequence pairs are encoded by corresponding subnetworks of the Siamese network. Their contrastive labels are used to construct the contrastive loss, while their individual labels are used to construct the classification loss.

**Contrastive loss of Siamese network**

Let $s_{i}$ and $s_{j}$ be two sequences, and $c_{i,j}\in\{0,1\}$ be their contrastive label, where $c_{i,j}=1$ if both of them are promoters or non-promoters, otherwise $c_{i,j}=0$. The Siamese network of our SiamProm makes sure that $s_{i}$ and $s_{j}$ are close if $c_{i,j}=1$ or they are distant if $c_{i,j}=0$ as much as possible. Aiming to this goal, we define the contrastive loss function $\mathcal{L}_{1}$ during the sequence representation phase:

|  | $\mathcal{L}_{1}=\frac{1}{K}\sum_{\begin{aligned} i,j=1 \\ i\neq j \end{aligned}}^{K} \left[ c_{i,j}{D\left( \mathbf{v}_{i},\mathbf{v}_{j} \right)}^{2}+\left( 1-c_{i,j} \right){\max\left( 0,m-D\left( \mathbf{v}_{i},\mathbf{v}_{j} \right) \right)}^{2} \right]$ | (10) |
| --- | --- | --- |

where $\mathbf{v}_{i}$ and $\mathbf{v}_{j}$ are the embeddings of $s_{i}$ and $s_{j}$, $D\left( \mathbf{v}_{i},\mathbf{v}_{j} \right)=\left\| \mathbf{v}_{i}-\mathbf{v}_{j} \right\|_{2}$ is the Euclidean distance between the two sequences, K is the total number of sequence pairs, and $m$ is a tunable margin distance [14].

**Binary predictor and classification loss**

After passing the Siamese network, the predictor, the last component of our SiamProm, treats sequences as samples $\{\mathbf{v}_{i}\}$. An MLP is implemented as the binary predictor $y_{i}^{*}=MLP\left( \mathbf{v}_{i} \right)\in[0,1]$, where $y_{i}^{*}$ is the predicted confidence score of being a promoter. It directly indicates how likely a given sequence $s_{i}$ is a promoter. Let $y_{i}\in\{0,1\}$ be the label of sequence $s_{i}$, where $y_{i}=1$ if $s_{i}$ is a promoter, $y_{i}=0$ otherwise. We employ the binary cross-entropy function to measure the label discrepancy (loss) between predicted labels and real labels as follows:

|  | $\mathcal{L}_{2}=-\frac{1}{M}\sum_{i=1}^{M} \left[ y_{i}\log\left( y_{i}^{*} \right)+\left( 1-y_{i} \right)\log\left( 1-y_{i}^{*} \right) \right]$ | (11) |
| --- | --- | --- |

**Parameter setting**

Like other DL-based models, our SiamProm contains model architecture parameters (e.g., embedding dimensions and numbers of hidden neurons) and learning hyperparameters (e.g., the learning rate, batch size, and optimizer momentum). We empirically set the architecture parameters while fine-tuning the learning hyperparameters by employing the Tree-structured Parzen Estimator (TPE) sampling method [15] and the Hyperband pruning algorithm [16] from the Optuna library [17]. Compared with the time-consuming greedy search, TPE sampling has a good global exploration capability to jump out local optimality, while the Hyperband pruning can discard bad parameter values to reduce computational time and resources significantly. The entire training process was performed under 10-fold cross-validation.

The detailed settings of the parameters are as follows. In the embedding initializer, we set $k=3$ to obtain the token set with $\left| V \right|=64$, such that the sequence with a length of $81$ bp is tokenized into a token sequence with a length of $79$ ($l^{*}=79$). In addition, the sequence is embedded into a $1024$-dimensional feature vector (i.e., $d^{e}=1024$). Furthermore, when performing positional encoding on the sequence, we introduce a $10\%$ dropout to decrease the model’s reliance on particular positions.

Among three parallel modules, the multi-head self-attention layer in the k-mer attention module contains four heads (i.e., $r=4$), and the dimensions of its Query, Key, and Value are all set to $64$ (i.e., $d_{k}=d_{v}=64$). In addition, its adaptor has $256$ input neurons. The Bi-LSTM in the bi-directional context catcher contains two hidden layers, where the dimension of each hidden layer is $32$ (i.e., $d_{l}=32$). The adaptor in this module contains $64$ input neurons. The 1D convolution layer in the nearest-neighbor aggregator has $1024$ channels (i.e., $d_{c}=1024$) with the kernel size=$3$. In addition, the padding technique is used to ensure the sequence length remains unchanged after convolution. Its adaptor contains $1024$ input neurons. The numbers of the output neurons of all the adaptors are set to $d_{e}$ because of residual connections. In addition, the size of all average pooling kernels in the pooling layer is equal to the length of the sequence.

In the compressor module, the long-concatenated sequence features are eventually mapped into a 128-dimensional sequence feature vector (i.e., $d=128$). We also establish that the margin distance $m=2$ in the contrastive loss function.

In the binary predictor, the predictor is equipped with $32$ hidden neurons and eventually outputs the probabilities for two categories.

Furthermore, we set the batch size to $256$ and the initial learning rate $\alpha$ as $2.2e-4$. We selected Adam as the optimizer with a decay rate of $0.9$ for the first moment and $0.99$ for the second moment. For faster model convergence, an early stopping mechanism was implemented by monitoring the metrics of the validation set. If a monitored metric remains un-updated for over ten epochs, the training process will be terminated.

**Supplementary Table**

**Table S1.** IUPAC-IUB codes.

| Symbol | Meaning | Origin of designation |
| --- | --- | --- |
| R | G or A | puRine |
| Y | T or C | pYrimidine |
| M | A or C | aMino |
| K | G or T | Keto |
| S | G or C | Strong interaction (3 H bonds) |
| W | A or T | Weak interaction (2 H bonds) |
| H | A or C or T | not-G, H follows G in the alphabet |
| B | G or T or C | not-A, B follows A |
| V | G or C or A | not-T (not-U), V follows U |
| D | G or A or T | not-C, D follows C |
| N | G or A or T or C | aNy |

**Table S2.** Performance comparison of SiamProm under different datasets generated by phantom sampling.

| Dataset | Acc (%) | Sn (%) | Sp (%) | MCC |
| --- | --- | --- | --- | --- |
| Dataset 1 | 89.13 ± 0.52 | 88.87 ± 1.33 | 89.38 ± 1.46 | 0.7825 ± 0.0232 |
| Dataset 2 | 88.77 ± 0.41 | 89.11 ± 1.19 | 88.42 ± 0.88 | 0.7754 ± 0.0196 |
| Dataset 3 | 88.69 ± 0.39 | 88.16 ± 1.22 | 89.22 ± 0.22 | 0.7738 ± 0.0187 |
| Dataset 4 | 89.05 ± 0.62 | 87.97 ± 1.58 | 90.13 ± 1.52 | 0.7812 ± 0.0251 |
| Dataset 5 | 88.36 ± 0.37 | 88.54 ± 0.92 | 88.54 ± 1.08 | 0.7672 ± 0.0174 |
| Ref Dataset | 88.74 ± 0.43 | 87.20 ± 1.21 | 90.30 ± 1.33 | 0.7754 ± 0.0205 |

**Table S3**. Abbreviation List

| **Abbreviation** | **Full name** |
| --- | --- |
| *E. coli* | *Escherichia coli* |
| *B. subtilis* | *Bacillus subtilis* |
| TSS | Transcription start site |
| ML-based | Machine learning-based |
| DL-based | Deep learning-based |
| CNN | Convolutional neural networks |
| RNN | Recurrent neural networks |
| CDS | Coding DNA sequence |
| SVM | Support vector machine |
| IND | Independent testing set |
| MLP | Multilayer perceptron |
| Acc | Accuracy |
| Sn | Sensitivity |
| Sp | Specificity |
| MCC | Matthew’s correlation coefficient |
| TP | True positive |
| TN | True negative |
| FP | False positive |
| FN | False negative |
| BERT | Bidirectional encoder representations from transformers |
| t-SNE | t-distributed stochastic neighbor embedding |
| w/o | Without |
| CRISPRs | Clustered regularly interspaced short palindromic repeats |
| REP | Repetitive extragenic palindromic |
| CKSNAP | Composition of k-spaced nucleic acid pairs |
| PseKNC | Pseudo k-tuple nucleotide composition |
| TPCP | Trinucleotide physicochemical properties |
| PWM | Position weight matrix |
| Bi-LSTM | Bi-directional long short-term memory |
| TPE | Tree-structured parzen estimator |

**Table S4**. Notation List

| **Symbol** | **Definition** |
| --- | --- |
| $\sigma^{N}$ | Different types of sigma factors |
| $d_{w}$ | Wasserstein distance |
| $d_{w}^{rand}$ | Wasserstein distance under random generation |
| $d_{w}^{part}$ | Wasserstein distance under partial substitution |
| $d_{w}^{CDS}$ | Wasserstein distance under CDS sampling |
| $d_{w}^{phan}$ | Wasserstein distance under phantom sampling |
| $d_{w}^{real}$ | Wasserstein distance under real non-promoters |
| $k$ | The k value in k-mer |
| $V$ | The k-mer token set |
| $l$ | An $l$-length nucleotide sequence |
| $s$ | Represent a nucleotide sequence |
| $s^{*}$ | A nucleotide sequence after tokenized |
| $\mathbf{S}$ | The matrix after the sequence is encoded |
| $t_{i}$ | One hot encoding of a row in matrix $\mathbf{S}$ |
| $\mathbf{H}^{e}$ | The initial token representation of $s$ |
| $\mathbf{W}_{s}$ | The weight matrix of $\mathbf{S}$ |
| $d_{e}$ | The dimension of token embeddings |
| $\mathbf{h}_{i}^{\mathrm{pos}}$ | Representation of $t_{i}$ after Positional encoding |
| $\mathbf{H}^{\mathrm{pos}}$ | Positional encoding matrix of the sequence $s$ |
| $\mathbf{H}$ | The initial token embedding of sequence $s$ |
| $\mathbf{Q}$ | Q matrix of attention module |
| $\mathbf{K}$ | K matrix of attention module |
| $\mathbf{V}$ | V matrix of attention module |
| $\mathbf{W}^{Q}$ | The weight matrix of $\mathbf{Q}$ |
| $\mathbf{W}^{K}$ | The weight matrix of $\mathbf{K}$ |
| $\mathbf{W}^{V}$ | The weight matrix of $\mathbf{V}$ |
| $\mathbf{H}^{\mathrm{attn}}$ | $\mathbf{H}$ after passing through the attention module |
| $\mathbf{v}^{\mathrm{attn}}$ | Sequence embedding after $\mathbf{H}^{\mathrm{attn}}$ passes through a pooling layer |
| $\mathbf{i}_{t}$ | The input gate |
| $\mathbf{f}_{t}$ | The forget gate |
| $\mathbf{o}_{t}$ | The output gate |
| $\mathbf{g}_{t}$ | The memory cells |
| $\mathbf{c}_{t}$ | The memory cell state |
| $\mathbf{h}_{t}$ | The input embedding |
| $\mathbf{z}_{t}$ | The hidden state |
| $\mathbf{W}_{\boldsymbol{*}}^{h}$ | The weight matrices in Bi-LSTM (for $\mathbf{h}_{t}$) |
| $\mathbf{W}_{\boldsymbol{*}}^{z}$ | The weight matrices in Bi-LSTM (for $\mathbf{z}_{t}$) |
| $\mathbf{b}_{\boldsymbol{*}}$ | The bias weight matrices in Bi-LSTM |
| $\sigma$ | The sigmoid function in Bi-LSTM |
| $⨀$ | The Hadamard product |
| $\mathbf{Z}_{f}^{\mathrm{lstm}}$ | Stacked hidden states in forward LSTM |
| $\mathbf{Z}_{r}^{\mathrm{lstm}}$ | Stacked hidden states in backward LSTM |
| $\mathbf{H}^{\mathrm{lstm}}$ | $\mathbf{H}$ after passing through the Bi-LSTM module |
| $\mathbf{v}^{\mathrm{lstm}}$ | Sequence embedding after $\mathbf{H}^{\mathrm{lstm}}$ passes through a pooling layer |
| $\mathbf{H}^{\mathrm{conv}}$ | $\mathbf{H}$ after passing through the convolution module |
| $\mathbf{W}^{a}$ | The learnable parameter from the adaptor |
| $\mathbf{W}^{c}$ | The weight matrix of the convolutional kernel |
| $\mathbf{b}^{c}$ | The bias term of the convolution |
| $\mathbf{v}^{\mathrm{conv}}$ | Sequence embedding after $\mathbf{H}^{\mathrm{conv}}$ passes through a pooling layer |
| $\mathbf{v}^{\mathrm{concat}}$ | Concatenation of $\mathbf{v}^{\mathrm{attn}}$, $\mathbf{v}^{\mathrm{lstm}}$, $\mathbf{v}^{\mathrm{conv}}$ |
| $\mathbf{v}$ | Dimensionality reduction of $\mathbf{v}^{\mathrm{concat}}$ |
| $\mathbf{W}^{a}$ | The weight matrix of the compressor |
| $\mathbf{b}^{a}$ | The bias of the compressor |
| $c_{i,j}$ | Contrastive label of sequences $s_{i}$ and $s_{j}$ |
| $\mathcal{L}_{1}$ | The contrastive loss function |
| $y_{i}$ | Sequence label (promoter or non-promoter) |
| $y_{i}^{*}$ | The predicted confidence score of being a promoter |
| $\mathcal{L}_{2}$ | Binary cross-entropy loss |
| $d^{e}$ | Embedding dimension |
| $r$ | The number of heads of attention module |
| $d_{k}$ | The column dimension of $\mathbf{K}$ |
| $d_{v}$ | $T\mathrm{he}\mathrm{column} dimension of \mathbf{V}$ |
| $d_{l}$ | The dimension of each hidden layer in Bi-LSTM |
| $d_{c}$ | The dimension of convolution layer |
| $m$ | Margin distance of contrastive loss |
| $\alpha$ | Learning rate |

**Supplementary Figure**

**Supplementary Figure S1.** Top 10 occurrences of 6-mer tokens. ‘CGATCG’, ‘GATCGC’, and ‘GCGATC’ always occur in an overlapping way, and they appear anywhere except in the -10 region.

**Supplementary Figure S2.** Top 2 ~ 9 occurrences of 8-mer tokens. They are highly similar to ‘GCGATCGC’, and their longest common sub-token, ‘CGATCG’ determined by multiple sequence alignment.

**References**

1. Hong Z, Zeng X, Wei L, et al. Identifying enhancer–promoter interactions with neural network based on pre-trained DNA vectors and attention mechanism. *Bioinformatics* 2020; **36**(4):1037–1043

2. Ji Y, Zhou Z, Liu H, et al. DNABERT: pre-trained Bidirectional Encoder Representations from Transformers model for DNA-language in genome. *Bioinformatics* 2021; **37**(15):2112–2120

3. Ju Z, Wang S-Y. Prediction of lysine formylation sites using the composition of k-spaced amino acid pairs via Chou’s 5-steps rule and general pseudo components. *Genomics* 2020; **112**(1):859–866

4. Chen W, Lei T-Y, Jin D-C, et al. PseKNC: a flexible web server for generating pseudo K-tuple nucleotide composition. *Anal Biochem* 2014; **456**:53–60

5. Feng P, Chen W, Lin H. Prediction of CpG island methylation status by integrating DNA physicochemical properties. *Genomics* 2014; **104**(4):229–233

6. Claverie JM, Audic S. The statistical significance of nucleotide position-weight matrix matches. *Comput Appl Biosci* 1996; **12**(5):431–439

7. Mikolov T, Chen K, Corrado G, et al. Efficient estimation of word representations in vector space. arXiv Preprint 2013; arXiv:1301.3781

8. Manavalan B, Basith S, Shin TH, et al. Computational prediction of species-specific yeast DNA replication origin via iterative feature representation. *Brief Bioinform* 2021; **22**(4):bbaa304

9. Taylor FJR, Coates D. The code within the codons. *Biosystems* 1989; **22**(3):177–187

10. Minchin S, Lodge J. Understanding biochemistry: structure and function of nucleic acids. *Essays Biochem* 2019; **63**(4):433–456

11. Vaswani A, Shazeer N, Parmar N, et al. Attention is all you need. *Advances Neural Inf Process Syst* 2017; 5998-6008

12. He K, Zhang X, Ren S, et al. Deep residual learning for image recognition. In: *Proceedings of the IEEE Conference on Computer Vision and Pattern Recognition* , Las Vegas, NV, USA: IEEE, 2016; 770–778

13. Takase S, Kiyono S, Kobayashi S, et al. On layer normalizations and residual connections in transformers. arXiv Preprint 2022; arXiv:2206.00330

14. Hadsell R, Chopra S, LeCun Y. Dimensionality reduction by learning an invariant mapping. In: *2006 IEEE Computer Society Conference on Computer Vision and Pattern Recognition (CVPR’06)*, 2006; 1735–1742. IEEE

15. Bergstra J, Bardenet R, Bengio Y, et al. Algorithms for hyper-parameter optimization. *Advances Neural Inf Process Syst* 2011; **24**

16. Li L, Jamieson K, DeSalvo G, et al. Hyperband: A novel bandit-based approach to hyperparameter optimization. *J Mach Learn Res* 2018; **18**(185):1–52

17. Akiba T, Sano S, Yanase T, et al. Optuna: A next-generation hyperparameter optimization framework. In: *Proceedings of the 25th ACM SIGKDD international conference on knowledge discovery & data mining* 2019; 2623–2631
